# Brucellosis-associated hemophagocytic lymphohistiocytosis: a case report and literature review

**DOI:** 10.3389/fmed.2025.1609644

**Published:** 2025-09-11

**Authors:** Shuwen Jiang, Binfeng Xia, Hongyu Wu, Peng Zhang, Xiaohua Li, Kaiyu Zhang

**Affiliations:** ^1^Department of Infectious Diseases and Center of Infectious Diseases and Pathogen Biology, The First Hospital of Jilin University, Changchun, China; ^2^Department of Gastroenterology, Lequn Branch, The First Hospital of Jilin University, Changchun, China

**Keywords:** brucellosis, hemophagocytic lymphohistiocytosis, pathogenesis, diagnosis, treatment

## Abstract

**Introduction:**

Hemophagocytic lymphohistiocytosis (HLH), a rare and deadly disease, is typically classified as either primary (familial) or secondary (acquired), depending on the etiology and underlying cause. Secondary HLH often develops in the presence of infectious, malignant, rheumatologic, or metabolic conditions, with infections, especially *Epstein–Barr virus* (EBV) infection, being among the leading causes. *Brucella* infection-induced HLH is relatively rare, with only eight cases reported in the past decade, all of which had a favorable prognosis following timely diagnosis and treatment.

**Case description:**

A 53-year-old man with brucellosis who developed secondary HLH and multiple organ dysfunction presented to our hospital with a 2-month history of fever and abnormal liver enzymes. Initial blood culture following admission confirmed *Brucella* spp. in the aerobic bottle after ⁓87.85 h of incubation. However, after the initial discharge, the patient did not adhere to the prescribed antibiotic therapy and subsequently developed symptoms of fever and abdominal discomfort, and was readmitted to our hospital. Laboratory examination also revealed pancytopenia. An additional blood culture further revealed the growth of *Brucella* spp. in the aerobic bottle after ⁓113.67 h of incubation. Other findings included decreased fibrinogen, increased ferritin, increased soluble IL-2 receptor *α* chain (sCD25), decreased Natural Killer (NK) cell activity, presence of hemophagocytic cells in the bone marrow smear, splenomegaly, and abnormal liver and kidney functions. The HScore score was 230 points. A thorough assessment was made, which led to the exclusion of other possible diseases, culminating in the identification of *Brucella* infection as the most probable cause of HLH. Consequently, the patient was given anti-infection (doxycycline, levofloxacin, etimicin, and rifampin), glucocorticoids (GCs), human immunoglobulin (HIG), and other symptomatic supportive treatments, which ultimately improved his condition.

**Conclusion:**

Despite the generally poor prognosis of HLH patients, those with Brucella-induced HLH may have a favorable outcome with prompt intervention. Conversely, a delayed treatment could increase the risk of HLH onset and progression, leading to death in severe cases.

## 1 Introduction

Hemophagocytic lymphohistiocytosis (HLH) is a life-threatening immune-mediated disease characterized by uncontrolled cytotoxic lymphocyte and macrophage activation, leading to cytokine-mediated tissue injury and multiorgan dysfunction (1). Clinical manifestations include fever, hepatosplenomegaly, pancytopenia, and the presence of activated macrophages in hematopoietic organs (2). According to reports, HLH has a high mortality rate ranging between 42% and 88% (1) and can occur at any age (3), with adult patients often having a poor prognosis. Furthermore, depending on the etiology and underlying cause, HLH could be categorized as primary (familial) or secondary (acquired). Secondary HLH could be attributed to infectious, malignant, rheumatologic, or metabolic conditions, with a high mortality rate ranging from 50% to 70% (4). In adults, viral infection has been identified as the most common trigger for acquired HLH, with herpes viruses, *Epstein–Barr virus* (EBV), and *Cytomegalovirus* (CMV) as the most commonly reported viral pathogens (1, 5). Chronic viral infections such as *human immunodeficiency virus* (HIV) and *hepatitis B* and *hepatitis C* may also trigger HLH during the acute or chronic infection phase (1). Furthermore, although rare, bacterial, parasitic, and fungal agents could cause HLH, with intracellular pathogens such as *Mycobacterium tuberculosis* (TB), *Pneumocystis jirovecii*, and *Plasmodium species* as the most widely reported (1, 5). Certain zoonotic diseases, including tick-borne illnesses and dengue fever, could also trigger secondary HLH. In a systematic review of 98 cases of tick-borne infection-associated HLH, Dorde Jevtic et al. (6) found that HLH secondary to tick-borne pathogens is relatively uncommon, with *Ehrlichia* spp. as the most frequently implicated tick-borne agent; however, the mortality rate in their study was 16.3%. Similarly, Leong Tung Ong and Roovam Balasubramaniam (7) reported that while HLH prevalence is low among dengue patients, dengue-associated HLH still carries a significant mortality risk, with a reported overall mortality rate of 20.2%. On the other hand, *Brucella* infection-induced HLH is quite rare, with only eight cases reported in the past decade, all exhibiting a good prognosis following timely diagnosis and treatment (8–15) (Table 1), thus highlighting the significance of early diagnosis and treatment for *Brucella* infection-associated HLH.

**Table 1:** Case reports of brucellosis complicated with hemophagocytic lymphohistiocytosis over the past 10 years.

Case	Career	Year/Sex	Clinical picture	*Brucella* species	Treatment	Outcome	References
1	Farmer	63 Y/F	High fever, dizziness, shivering, incontinence, and blurring of consciousness	*Brucella melitensis*	Doxycycline, Rifampicin, and Moxifloxacin	Improvement of clinical symptoms	(8)
2	Not available	37 Y/M	Dizziness, fatigue, nausea, vomiting, and joint pain for 10 days and fever for 5 days	Not identified	Doxycycline, Rifampicin, Sulfamethoxazole, Dexamethasone (17 mg/d), and Etoposide (250 mg/d)	8 weeks later, the patient returned to normal	(9)
3	Not available	60 Y/M	Fevers, chills, night sweats, and a 50-to-20-pound weight loss for approximately 3 months	*Brucella melitensis*	Doxycycline, Rifampin, and Dexamethasone (10 mg/m^2^/d)	Improvement of clinical symptoms	(10)
4	Not available	16 Y/F	Prolonged fever, weight loss, arthralgia, loss of appetite, and fatigue	Not identified	Rifampicin and Doxycycline Intravenous immunoglobulin (400 mg/kg/d for 1 day), Dexamethasone (10 mg/m^2^/d for 2 weeks; thereafter the dosage was reduced by 50% every week and discontinued at the end of 6 weeks)	6 weeks later, the patient returned to normal	(11)
5	Farmer	73 Y/M	Fever, loss of appetite, and lumbar pain for 10 days	*Brucella melitensis*	Doxycycline, Rifampicin	After 10 days of treatment, the laboratory test results and clinical symptoms were improved	(12)
6	Not available	4 Y/M	Fever, myalgia, and appetite loss for 3 weeks	Not identified	Doxycycline and Trimethoprim Sulfamethoxazole (TMP-SMX)	The child showed complete clinical and biochemical improvement after 6 weeks of therapy	(13)
7	Not available	50 Y/M	Fever and weakness for 6 days and diarrhea for half a day	*Brucella melitensis*	Doxycycline, Cefoperazone/Sulbactam	The laboratory data of day 10 showed recovery.	(14)
8	Not available	31 Y/M	Generalized fatigability, anorexia, lassitude, and mood disturbance and fever lasting for 1 weeks	Not identified	Doxycycline, Rifampicin	The laboratory data returned to normal after 6 weeks of therapy.	(15)

Clinical information of brucellosis cases complicated with hemophagocytic lymphohistiocytosis.

Brucellosis is a pervasive zoonotic disease caused by various Brucella species (16). The *Brucella* genus comprises six species, each named after its principal host: *B. melitensis* (sheep and goats), *B. abortus* (cattle), *B. suis* (pigs), *B. ovis* (sheep), *B. canis* (dogs), and *B. neotomae* (wood desert rats). Notably, although the disease’s incubation period typically ranges from 2 to 4 weeks, it could last several months (16, 17). Additionally, according to a 2006 report, ⁓500,000 new cases of brucellosis are diagnosed globally each year (18) and contact with infected animals (e.g., cattle and sheep) and consumption of contaminated animal products have been established as the primary transmission pathways for the disease (19). Furthermore, its most common clinical symptoms include fever, fatigue, and liver and spleen enlargement. Moreover, the non-specific clinical symptoms of brucellosis could lead to a delayed diagnosis, allowing the disease to progress to HLH.

Herein, we systematically reviewed a brucellosis-induced HLH case and related HLH literature through a comprehensive search of the PubMed medical database (Search query: “Brucellosis” AND “Lymphohistiocytosis, Hemophagocytic”[Mesh] or “hemophagocytic lymphohistiocytosis”). We also cross-referenced existing review articles to obtain other relevant reports.

## 2 Case description

A 53-year-old man presented to our hospital with a 2-month history of fever and abnormal liver enzymes. Notably, the patient was a sheep breeder and had a prior history of contact with sheep. Blood samples were drawn from the patient into a Mérieux blood culture bottle and incubated at 35 °C for 5 days, and the initial blood culture (aerobic) revealed the presence of *Brucella*. Mass spectrometry also confirmed the presence of *Brucella* in the culture. Notably, due to inappropriate laboratory conditions, we could not identify the species of the *Brucella* genus. The patient was discharged after some time with Doxycycline and Levofloxacin prescriptions for oral antimicrobial therapy. However, the patient did not take the antibiotics as prescribed. Within 4 days, he presented to a local hospital again with complaints of fever and abdominal discomfort, as well as pancytopenia (the specific value was unknown). The patient was later re-admitted to our hospital following a platelet transfusion and bone marrow aspiration. Notably, the patient had a 2-month history of diabetes mellitus (DM) without diabetic complications. The glycosylated hemoglobin concentration 2 months before admission was 7.2% (NR:4.0%–6.0%) but did not receive systemic treatment. He also denied a history of other illnesses or a familial genetic history of specific diseases.

Upon admission, the preliminary physical examination revealed a temperature of 38.6 °C, a respiration rate (RR) of 26 breaths/min, a pulse of 88 beats/min, a blood pressure (BP) of 99/68 mmHg, a height of 168 cm, and a weight of 60 kg. The patient had no jaundice of the skin or sclera. The cardiopulmonary assessment revealed no abnormalities. The abdominal examination revealed mild tenderness in the right upper quadrant without rebound pain or rigidity. The liver and spleen were not palpable below the ribs. The lower limbs showed no edema.

**Figure 1:**
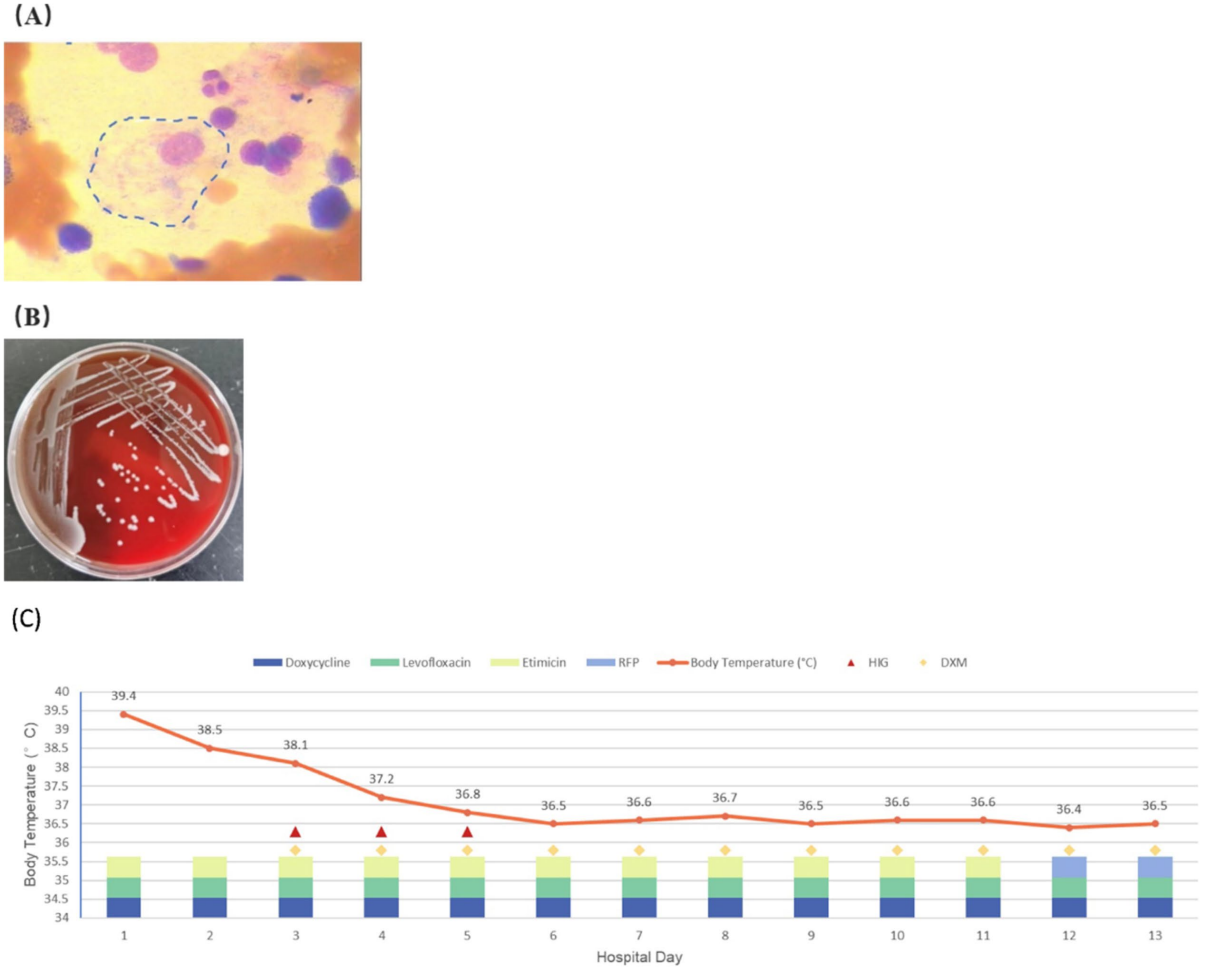
**(A)** Hemophagocytic cells are observed in the patient’s bone marrow smear. **(B)** Blood aerobic culture positive for *Brucella*. **(C)** Temperature changes and the treatment of the patient during hospitalization.

Table 2 details the main laboratory test results during hospitalization and other related examinations. Routine coagulation tests revealed an activated Partial Thromboplastin Time (APTT) of 51.3 s (normal range [NR]: 21–33 s). Furthermore, the fasting triglyceride (Ftriglyceride), cholesterol, high-density lipoprotein cholesterol (HDL-C), and low-density lipoprotein cholesterol (LDL-C) levels were 2 mmol/L(NR:0.28–1.8 mmol/L), 2.53 mmol/L (NR:2.6–6 mmol/L), 0.41 mmol/L(NR:0.76–2.1 mmol/L), and 1.62 mmol/L(NR:2.06–2.1 mmol/L), respectively. The glycosylated hemoglobin value at 2 months before admission was 7.2% (NR:4.0%–6.0%). A bone marrow smear identified histiocytes with occasional hemophagocytic cells (Figure 1A). On day 6 of hospitalization, a blood culture confirmed the growth of *Brucella* spp. in an aerobic bottle after ⁓113.67 h of incubation (Figure 1B). On day 8 of hospitalization, the blood culture revealed the growth of *Brucella* spp. in an aerobic bottle after ⁓94.87 h of incubation. Antinuclear antibody (ANA) profiles, antiplatelet antibodies, CMV, EPV, hepatitis B, hepatitis C, syphilis, and HIV were all negative.

**Table 2:** Major laboratory test results of the patient during hospitalization.

Test item	Before treatment	At discharge	Follow up for 8 weeks	Normal range
WBC count	2.94	4.17	Normal	3.50–9.50 × 10^9^/L
HGB	79	88	Normal	130–175 g/L
PLT count	17	45	Normal	125–350 × 10^9^/L
Aspartate aminotransferase	116.7	33.2	Not detected	15–40 IU/L
Alanine aminotransferase	53.6	19.3	Not detected	9–50 IU/L
Creatinine	183.9	66.3	Not detected	58–110 μmol/L
Lactate dehydrogenase	416	242	Not detected	120–250 U/L
Fibrinogen	1.1	2.36	Not detected	1.8–4.0 g/L
Ferroprotein	5,528	934	Not detected	23.9–336.2 μg/L
sCD25	43,281	Not detected	Not detected	< 6,400 pg./mL
NK cell activity	13.95	Not detected	Not detected	≥ 15.11%

WBC, white blood cell; HGB, hemoglobin; PLT, platelet; NK, natural killer; sCD25, soluble IL-2 receptor α chain.

Regarding the imageological examination, aortic valve calcification was the key cardiac ultrasound finding. Meanwhile, the ultrasound findings of the digestive system included a fatty liver, hepatomegaly, gallbladder wall edema, splenomegaly, hypoechoic splenic lesions, and ascites.

The patient was diagnosed with brucellosis 2 months ago and put on doxycycline and levofloxacin for 1 week. However, he did not continue taking either prescription post-discharge. Fever was the main symptom during the patient’s second hospitalization. Based on the above laboratory findings, the patient met the HLH-2004 diagnostic criteria (20) (Table 3), with an HScore (21) of 230. Blood cultures detected *Brucella* spp. in aerobic bottles, leading to the diagnosis of secondary hemophagocytic lymphohistiocytosis (HLH) induced by Brucella infection. The patient received systemic anti-infective therapy, glucocorticoids, and intravenous immunoglobulin (IVIG). The specific dosages and treatment duration were as follows: The specific antibiotic treatment regimen was: doxycycline (100 mg every 12 h via Intravenous [IV] infusion during hospital days 1 to 5 and 100 mg administered orally every 12 h during hospital days 6 to 13); levofloxacin (200 mg every 24 h through IV infusions with the initial dose doubled during hospital days 1 to 4 and 200 mg every 12 h through IV during hospital days 5 to 13); etimicin (300 mg through IV every 24 h during hospital days 1 to 7, 200 mg through IV every 24 h during hospital days 8 to 10, and 300 mg through IV every 24 h in hospital day 11), and rifampin (600 mg administered orally every 24 h during hospital days 12 to13). Dexamethasone (15 mg/day from hospital days 3 to 11, then 10 mg/day from days 12 to 13). Intravenous immunoglobulin (IVIG) (250 mg/kg/day from hospital days 3 to 5).

**Table 3:** HLH-2004 diagnostic criteria.

Diagnostic criteria	
1	Fever: body temperature > 38.5 °C for >7 days
2	Splenomegaly
3	Cytopenias (affecting ≥ 2 of 3 lineages in the peripheral blood); hemoglobin < 90 g/L (< 4 weeks infants, hemoglobin < 100 g/L); platelets < 100 × 10^9^/L; neutrophils < 1.0 × 10^9^/L
4	Hypertriglyceridemia and/or hypofibrinogenemia: fasting triglycerides (≥ 3.0 mmol/L or ≥ 265 mg/dL); fibrinogen ≤ 1.5 g/L
5	Ferritin ≥ 500 μg/L
6	Hemophagocytosis in bone marrow, spleen, or lymph nodes; no evidence of malignancy
7	Low or absent NK-cell activity (according to local laboratory reference)
8	Soluble CD25 (or soluble IL-2 receptor) ≥ 2,400 U/mL

Diagnosis of HLH requires at least five of the listed eight features. HLH, hemophagocytic lymphohistiocytosis; NK, natural killer.

During the early hospitalization period, the patient showed no significant improvement following initiation of antimicrobial therapy in clinical symptoms or laboratory parameters, including complete blood count, ferritin, and fibrinogen levels. Consequently, the treatment regimen was optimized by the inclusion of glucocorticoids and intravenous immunoglobulin (IVIG) in ongoing antimicrobial therapy. This combined therapeutic approach caused a significant clinical improvement relative to the status at admission, with significant normalization of hematological indices (complete blood count), ferritin, and lactate dehydrogenase (LDH) levels at the time of discharge. The patient achieved clinical stability by hospital day 13, and his fever curve and details of his therapeutic regimen during hospitalization are shown in Figure 1C. He was subsequently discharged. Post-discharge medication included rifampin, doxycycline, levofloxacin, and oral dexamethasone (10 mg/day). Complete recovery was achieved after 8 weeks of follow-up monitoring.

## 3 Discussion

Despite its significant clinical implications, the pathogenesis of HLH remains incompletely unclear. However, the disease has been largely associated with a significant reduction or obstruction of cytotoxic T cells and NK cytotoxic activity, resulting in ineffective clearance of viruses and other antigens. This immune dysfunction, combined with the persistent abnormal continuous activation of macrophages, can trigger a hyperinflammatory response in multiple organs, ultimately leading to immune-mediated tissue and organ damage (22). Under normal physiological conditions, CD8+ cytotoxic T cells and NK cells often release cytolytic granules containing perforin and granzyme when they come into contact with virus-infected or tumor cells. Herein, we reviewed a case of *Brucella* infection-induced secondary HLH. The resulting inability to clear antigen stimulation could, in turn, lead to immune response persistence and expansion. Activated immune cells have been established to release proinflammatory cytokines (IFN-*γ*, TNF-*α*, IL-1, IL-6, TNF-α, and so on), leading to high levels of macrophage activation, which, in turn, might cause hemophagocytosis, tissue damage, organ failure, and other inflammatory manifestations (23, 24). Relevant retrospective studies have shown that adult HLH is largely attributable to malignant tumors. Infections and autoimmune diseases are the other common causes of the disease, with viral infections, including EBV, HIV, CMV, or influenza, as the most common pathogens. Meanwhile, bacterial infection-induced HLH is relatively rare (25–27). Moreover, although *Brucella*—an intracellular bacterium that can manipulate host cell processes, disrupt phagocyte function, inhibit phagocytosis, and prevent host cell apoptosis—can cause secondary HLH, the underlying mechanisms remain underexplored.

*Brucella* subverts signaling pathways crucial for innate immunity, thus modulating the host immune response (16). Nonetheless, the mechanism underlying brucellosis-induced HLH remains unclear. Some researchers linked the hemophagocytic mechanism of non-viral infection to the excessive production of activating cytokines (such as TNF-*α* and interferon-*γ*), leading to macrophage activation. However, it could also be the result of poorly regulated or inappropriate T-helper lymphocyte responses to intracellular pathogens (28). Consequently, following *Brucella* infection, the host immune response produces numerous cytokines, leading to phagocytic cell activation and helper T lymphocyte dysfunction, ultimately inducing HLH. In this regard, it is noteworthy that the timely removal of bacteria, inflammation reduction, and immune response regulation would be particularly important interventions.

We compared and analyzed eight cases of hemophagocytic syndrome caused by Brucella infection (Table 1), which showed that the clinical manifestations and laboratory findings of brucellosis were comparable to those of HLH, especially in terms of hematologic abnormalities. Data show that *Brucella* species often replicate in host phagocytes, impairing immune responses (29). This phenomenon suggests that the patients’ blood system is often affected. Analysis of blood cells in 484 patients with brucellosis confirmed the presence of anemia (21.5%), thrombocytopenia (18.8%), leukopenia (14.6%), and pancytopenia (2%–14%) (30). Another study reported pancytopenia in 14.8% of 54 children with brucellosis (31). Furthermore, the pathogenesis of brucellosis-induced pancytopenia included the following: (1) Hemophagocytosis, (2) hypersplenism, (3) bone marrow dysgenesis, (4) bone marrow granuloma, and (5) immune destruction (32). Common histopathological manifestations of HLH include hemophagocytosis in the bone marrow and reticuloendothelial organs (5). Furthermore, complete blood count analyses in patients with HLH commonly reveal leukocytosis, anemia, thrombocytopenia, and elevated inflammatory markers such as C-reactive protein (CRP) and erythrocyte sedimentation rate (ESR). Given these findings, the presence of Brucella-induced pancytopenia should prompt clinical consideration of HLH as a potential diagnosis.

Several measures have been developed for assessing HLH. Henter et al. developed the HLH2004 criteria (Table 3) whereby a diagnosis of HLH is confirmed if five out of the eight criteria are met (20). Additionally, Fardet et al. (21) developed the HScore^1^–a total sum of the score of nine variables that allow for the assessment of HLH risk (Table 4) (27). Unlike the HLH-2004 criteria, which comprise parameters derived from a pediatric population, the HScore was developed in an adult cohort, including patients aged ≥ 18 years (33). Furthermore, the pattern of inflammatory cytokines (IFN-*γ* and IL-10 upregulation, with only modestly elevated IL-6 levels) demonstrated a high diagnostic accuracy for secondary hemophagocytic lymphohistiocytosis secondary hemophagocytic lymphohistiocytosis (sHLH) and could be useful in differentiating infection-induced HLH and monitoring patients (27).

**Table 4:** Parameters and points in the HScore (27).

Parameter	No. of points (criteria for scoring)
Known underlying immunosuppression^a^	0 (no) or 18 (yes)
Temperature (°C)	0 (<38.4), 33 (38.4–39.4), or 49 (>39.4)
Organomegaly	0 (no), 23 (hepatomegaly or splenomegaly), or 38 (hepatomegaly and splenomegaly)
No. of cytopenias^b^	0 (1 lineage), 24 (2 lineages), or 34 (3 lineages)
Ferritin (μg/L)	0 (<2,000), 35 (2,000–6,000), or 50 (>6,000)
Triglyceride (mmol/L)	0 (<1.5), 44 (1.5–4), or 64 (>4)
Fibrinogen (g/L)	0 (>2.5) or 30 (≤2.5)
Aspartate aminotransferase (U/L)	0 (<30) or 19 (≥30)
Hemophagocytosis on bone marrow aspirate	0 (no) or 35 (yes)

a HIV positive or receiving long-term immunosuppressive therapy (i.e., glucocorticoids, cyclosporine A, and azathioprine).

b Defined as a hemoglobin level of 9.2 g/L and/or a leukocyte count ≤ 5 × 10^9^/L and/or a platelet count ≤ 110 × 10^9^/L.

Our patient had positive blood and bone marrow cultures for *Brucella*. Meanwhile, the patient exhibited common clinical manifestations of brucellosis, including fever, splenomegaly, abnormal liver function, and elevated LDH levels, confirming the diagnosis of *Brucella*. Moreover, due to the presence of pancytopenia, especially thrombocytopenia, a bone marrow smear examination was performed, revealing hemophagocytic cells. Additionally, bone marrow aspiration was performed, revealing hemophagocytic cells. Based on these findings, along with laboratory ferritin, fibrinogen, soluble CD25, NK cell activity, and other test results, HLH diagnosis was confirmed.

Presently, the specific treatment for *Brucella*-induced HLH remains unknown, with most of the current interventions being empirical clinical treatments. The majority of HLH patients are currently being treated with antibiotic medications based on the HLH-2004/HLH-1994 protocol and La Rosée et al.’s recommendations for managing HLH in adults (hereinafter referred to as *the recommendations*) (20, 27, 34). *The recommendations* propose that the heterogeneity of adult HLH prohibits a “1-size-fits-all protocol” and that treatment should be tailored to control hyperinflammation and treat identified disease triggers, especially the disease-causing pathogen (*Brucella*). Qureshi et al. proposed doxycycline and RIF for treating brucellosis and minimizing the risk of relapse, with aminoglycosides frequently added during the initial 2–3 weeks of therapy (16, 35). While the WHO-recommended guidelines for brucellosis treatment have evolved, the optimal approach remains unclear (16). Moreover, important organs such as the liver and kidneys should always be protected during treatment. Therefore, appropriate antibiotics should be chosen for the whole course of treatment based on the patient’s organ function. Considering the aforementioned clinical guidelines and the severity of the patient’s condition, we initiated an antimicrobial therapy encompassing doxycycline, levofloxacin, and etimicin, with appropriate dosage adjustments based on the patient’s hepatic and renal function. Following a comprehensive treatment including the aforementioned antimicrobial agents, corticosteroids, and Intravenous Immunoglobulin (IVIG), the patient’s general condition and previously abnormal laboratory parameters improved significantly compared to admission status. Nonetheless, aerobic blood cultures obtained on hospital day 8 revealed the growth of Brucella species after ⁓94.87 h of incubation. Given the normalized liver function, we modified the antibiotic regimen to doxycycline, levofloxacin, and rifampicin to consolidate anti-infective therapy. Notably, some case reports indicated recovery from the disease with antibiotics alone (8, 12–15). On the other hand, the treatment drugs per the HLH-2004/HLH-1994 protocol and *the recommendations* mainly included GCs, immunosuppressants, biologic agents, and cytotoxic drugs. The main GCs were DEX (10 mg/m^2^/d for 2 weeks; thereafter reduced by 50% every 2 weeks and continued to the end of 8 weeks). In the majority of secondary HLH cases, the dosage and course of DEX should be stopped after controlling the primary disease per the clinical situation. Moreover, immunosuppressive agents and cytotoxic drugs may not be required when GCs are effective. The treatment of *Brucella*-associated HLH currently follows the principle of individualization. In this regard, it is noteworthy that intravenous immunoglobulin therapy has been successfully used in adults with multifactorial HLH. Two small series of adult HLH patients who received intravenous immunoglobulins exhibited promising results—especially those with HLH attributed to infections and autoimmune diseases—with an estimated survival rate (SR) of 59%–75% (36, 37). Furthermore, among the eight case reports from the past decade (Table 1), antibiotic therapy alone was effective in five patients, with the remaining three patients treated with the corticosteroid DEX in combination with anti-brucellosis therapy (HIG or etoposide in two cases). According to *the recommendations*, although patients infected by pathogens targeting the monocyte–macrophage system may develop HLH, immunosuppression as recommended in the HLH-94 protocol should be avoided, as they usually respond well to specific antimicrobial treatments. However, our patient’s cell count was not restored to normal after antimicrobial therapy with doxycycline, levofloxacin, and etimicin. Moreover, the platelet count and hemoglobin levels did not increase significantly following platelet transfusion. La Rosée et al. (27) proposed that IVIG could inhibit complement activation, antibody Fc fragments, and macrophage Fc receptors, as well as neutralize pro-inflammatory cytokines, thus exerting an anti-inflammatory effect. Considering the HLH-1994/HLH-2004 protocol and *the recommendations*, as well as the patient’s clinical status, we initiated treatment with corticosteroids and IVIG for the patient. Notably, the use of IVIG is quite debatable. The HLH-2004 protocol recommends IVIG at 0.5 g/kg once every 4 weeks (during initial and continuation therapy). On the other hand, La Rosée et al. (27) reported that IVIG should be administered over 2–3 days to a total dose of 1.6 g/kg. Furthermore, Georgia Griffin et al. (1) in their review of hemophagocytic syndrome-related literature, mentioned that Rong-Rong He et al. (38) reported comparable efficacy with regimens of 400 mg/kg/day for 5 days (IVIG5) and 1 g/kg/day for 2 days (IVIG2). Due to our patient’s financial constraints, the aforementioned doses could not be administered. However, the patient’s general condition and laboratory parameters improved after 3 days of alternative therapy. A dose of 250 mg/kg/day was given for 3 days, which represents a limitation as it is not the standard recommended dosage. Nonetheless, this treatment inhibited cellular inflammatory factor release and reduced phagocytic cell activation. The patient’s temperature was also restored to normal, while the white blood cell (WBC) count increased, and the hemoglobin and platelet levels remained stable without blood transfusion support. Based on these findings, we deduced that when treatment of *Brucella-induced* HLH with antibiotic therapy alone is not effective, timely application of GCs, such as DEX, along with HIG could shorten the treatment time.

Viral infection is the most common cause of adult HLH, with *EBV* being the most predominant pathogen. Although the timely initiation of the HLH-94 regimen could significantly improve the prognosis of EBV-HLH, the treatment intensity and duration should be stratified based on disease severity. For milder cases or patients with clinical improvements, a short-course corticosteroid regimen (with/without IVIG) could be a sufficient conservative approach. Nonetheless, etoposide must be administered immediately in cases of rapid clinical deterioration—particularly in untreated EBV-infected patients. Furthermore, while rituximab could eliminate *EBV* reservoirs in EBV-triggered HLH, it should not replace GC-based therapy (with/without etoposide) (27). Trym Fauchald et al. (39), in their study of TB-associated HLH, concluded that anti-mycobacterial therapy (with or without etoposide) could be a critical determinant of survival, with an overall survival (OS) rate marginally above 50%.

A summary of eight reports on cases of brucellosis-associated HLH (Table 1) was provided, which showed that the patients presented with diverse non-specific symptoms. Moreover, some patients lacked a clear epidemiological history associated with brucellosis. Fever was the main clinical manifestation (which lasted from a few days to several months), and was sometimes accompanied by splenomegaly. The common laboratory findings included cytopenia and increased ferritin levels, accompanied by abnormal liver function and LDH levels. In certain patients with prolonged fever, delayed identification of the underlying cause and the absence of timely intervention may contribute to the development of HLH. Notably, both the previously reported cases and the present case demonstrated marked improvement in clinical symptoms and laboratory findings following comprehensive and systematic treatment. Anti-infective therapy was the most effective treatment, and all patients received a standardized anti-infective regimen. Some patients improved with anti-infective therapy alone, while others required additional corticosteroids (with or without etoposide/human immunoglobulin) on top of the anti-infective treatment. Therefore, patients with prolonged fever and laboratory tests showing cytopenia, splenomegaly, and elevated ferritin levels should be actively screened for *Brucella* infection despite the absence of an epidemiological history of brucellosis. Meanwhile, further examinations (triglycerides, fibrinogens, marrow aspiration, sCD25, and NK cell activity) should be conducted to confirm the presence of HLH. In conclusion, early diagnosis is recommended to improve the prognosis of HLH patients.

This study highlights the fact that *Brucella*-induced HLH is rare. Furthermore, when encountering a patient with clinical manifestations such as fever, splenomegaly, and liver injury, clinicians should carefully consider the patient’s medical history, as well as the possibility of *Brucella* infection. Additionally, more laboratory tests should be conducted in cases displaying pancytopenia to confirm HLH diagnosis. Overall, early diagnosis and timely treatment can reduce disease progression and mortality rates in HLH patients, and patients with a confirmed *Brucella* infection should undergo regular antimicrobial therapy to prevent disease progression.

## Data Availability

The original contributions presented in the study are included in the article/supplementary material, further inquiries can be directed to the corresponding author.

## Footnotes

^1^ http://saintantoine.aphp.fr/score/
